# The double banality of evil in public health: institutional protocols and psychic numbing in the COVID-19 pandemic

**DOI:** 10.3389/fpubh.2026.1770965

**Published:** 2026-02-12

**Authors:** José A. Martínez

**Affiliations:** Department of Business Economics, Technical University of Cartagena - Member of European University of Technology, Cartagena, Spain

**Keywords:** banality of evil, COVID-19, ethical memory, invisible victims, psychic numbing

## Introduction

The COVID-19 pandemic left profound biological, social, and moral scars. In Spain, two groups remain on the margins of the official narrative: the 7,291 older adults who died in care homes in the Madrid region during the first wave without being transferred to hospital (1), and the citizens who reported severe side effects from vaccinations, which were frequently administered under perceived obligation (2, 3).

Beyond their suffering, these groups illustrate what this article refers to as a double banality of evil in the public health sphere. Drawing on Arendtian theory and contemporary moral psychology, the essay examines how systemic mechanisms render certain victims invisible. Hannah Arendt (4) described how ordinary people, sheltered by procedural compliance, can become involved in immense atrocities. This is not spectacular evil, but rather the gray evil of the efficient bureaucrat.

This essay argues that these victims have undergone a dual process of banalisation. The first is institutional banalisation, in which their lives are managed through bureaucratic protocols that render decision-making opaque and complex, thereby reducing accountability. The second is social banalisation, where their suffering is absorbed into public consciousness as statistical mass, a phenomenon known as the “psychic numbing” effect (5), which decreases sustained empathy. Public health cannot accept technical efficiency at the expense of moral memory, and this lens helps to highlight ethical failures.

## Institutional banalisation: protocols, denial, and legal impunity

Arendt observed that modern evil takes the form of impersonal processes in which moral responsibility is lost within protocols. In Madrid's care homes, this manifested as decisions that profoundly affected individuals, but were taken as administrative formalities.

The case is extensively documented. Between March and April 2020, at least 9,470 resident deaths were recorded, of which 7,291 (approximately 77%) occurred without hospital transfer (1). Pre-existing structural factors were compounded by specific political decisions, including “non-referral” protocols for certain clinical profiles (6). The institutional response has been characterized by systematic denial and legal impunity. As of 2025, at least 143 criminal cases had been dismissed, with courts validating the thesis that “everything humanly possible was done” (7). The Supreme Court further ruled that the regional government had no legal obligation to medicalize care homes, merely to establish guidelines (8). This situation can be interpreted as a form of institutional banalization. Not because the courts evaluated the substantive adequacy of the decisions taken, but because framing them as matters of administrative discretion in an emergency context effectively limited their legal accountability, rendering potentially preventable deaths non-justiciable.

A comparable logic applies to those reporting severe vaccine adverse effects. The core problem is not the existence of risks but the extreme difficulty in obtaining recognition and redress within a system that consistently communicated these medical products as unequivocally “safe and effective” (9). This construction of an official narrative functions as a bureaucratic filter that prevents individual claims of harm by framing them as threats to public confidence rather than as clinical realities. This creates a fundamental contradiction: when individuals experience adverse events, they encounter a system that perceives them not as persons with complex clinical histories, but as files that must fit narrow diagnostic categories. Furthermore, unlike countries such as Denmark, France, or the United Kingdom (10), Spain established no specific no-fault compensation system for COVID-19 vaccines, despite state liability through indemnity clauses with pharmaceutical companies. The first compensation ruling for a vaccine-injured patient did not occur until May 2024, with others remaining exceptional. The institutional language of population risk, whilst communicating epidemiological truths, relegates individual suffering to statistical insignificance against a backdrop of official certainty.

## Social banalisation: psychic numbing and the attenuated public response

The second banalisation occurs not within the halls of power, but in the sphere of public consciousness. It is here that the psychic numbing described by Slovic (5) takes hold, where the mind becomes anesthetized by mass suffering. The tragedy of the care homes was absorbed into the public discourse as abstract numbers (e.g., 53% excess mortality, 7,291 deaths), a statistical framing that, whilst epidemiologically necessary, inherently dampens emotional responses. The identifiable victim effect, which shows that a single, identifiable individual elicits far greater empathy than a statistical abstraction, was systematically neutralized.

This social banalisation was compounded by official denial. When the regional president of Madrid publicly dismissed the documented death toll as “an invention” (11), it actively eroded the social reality of the harm, creating a narrative fog that further discouraged public engagement and solidarity. The result was an attenuated social response; the scale of the tragedy never triggered a sustained and massive societal demand for accountability commensurate with the number of lives lost. Similarly, for vaccine adverse effects, the relentless public messaging of safety and efficacy created a cognitive environment where individual claims of harm were often perceived not as tragic exceptions but as threats to a collective salvation narrative, thereby limiting the emergence of broad public advocacy. This dynamic reflects a form of Tocqueville's “Tyranny of the Majority,” where the narrative of the surviving majority silences the minority. The suffering of the few is actively suppressed by the *argumentum ad populum* of collective success, rendering their reality invisible within the public sphere.

## The double banality: an integrated framework

The double banality of evil helps to link these two dynamics rather than treating them as separate problems. Institutional banalization—through protocols, legal impunity, and denial—creates the factual and narrative conditions for social banalization. By blocking official recognition and reducing victims to statistics or disputed figures, institutions deprive the public sphere of the concrete, identifiable stories that normally sustain empathy and moral outrage.

Conversely, social banalization, through psychic numbing and a muted collective response, creates a permissive environment in which institutional banalization can persist at little political cost. The absence of strong public pressure allows impunity and denial to stand. In the case of care homes, this meant residents disappeared twice: first from hospital wards because of protocols, and then from public discourse through abstraction and denial. For those affected by vaccines, it meant being invisible first as an exception to the safety narrative within healthcare, and then as a statistically negligible outlier in the public imagination.

## Conclusion: public health's debt to its victims

Raising these issues does not negate the complexity of pandemic management. Precisely for this reason, ethical reflection is even more necessary: the greater the appeal to the greater good, the more care is required in treating those who pay a disproportionate cost.

A mature public health system must assume three basic obligations if it is to break this cycle of banalization. First, an obligation to memory, actively preserving and making visible the names and stories behind the statistics. Second, an obligation of recognition, with formal channels for institutional listening and apology, independent of the outcome of litigation. Third, an obligation to reparation, developing fair mechanisms of compensation that acknowledge the specific nature of harm arising from public health interventions.

Public health cannot renounce numbers, but it must not allow numbers to erase lives. The idea of a double banality of evil offers a critical tool for identifying and resisting the systemic mechanisms that lead to victim invisibilization. It also sheds light on a shift in the political sphere, where scientific discourse was frequently mobilized as a source of unquestionable authority, displacing ethical deliberation and enabling political decisions to be presented as technical inevitabilities. By deliberately reconnecting the statistic with a face and the protocol with conscience, public health can begin to honor its debt to all victims and lay a more resilient and genuinely ethical foundation for future crises.
